# Water spirits within the fishers’ worldview: implications for fishing management in Northeast Brazil

**DOI:** 10.1186/s13002-019-0350-z

**Published:** 2019-12-23

**Authors:** André Bastos da Silva, João Batista Lopes, Luciano Silva Figueiredo, Roseli Farias Melo de Barros, Wedson Medeiros Silva Souto, Nelson Leal Alencar, Clarissa Gomes Reis Lopes

**Affiliations:** 1 Development and Environment Graduate Program, Federal University of Piauí (UFPI), Universitária, Teresina, Piauí 64049-550 Brazil; 2 Nature Science Center, State University of Piauí (UESPI), 25 Highway BR230, Picos, Piauí 64600-000 Brazil; 3 Department of Biology, UFPI, Teresina, Brazil

**Keywords:** Artisanal fishing, Local traditional knowledge, Symbology of water, Animism, Myth, Conservation

## Abstract

**Background:**

Historically, aquatic environments are linked to the worldview of many local people, where there is an interconnection between the natural world, the supernatural, and the social organization. In this study, we provided a discussion on how the supernatural beings that inhabit the freshwater systems interact with artisanal fishers and fishing resources in the riverine community of Parnaíba River middle course, in Mid North of Northeast Brazil. We also provided the implications of these interactions on the fisher’s behaviors and how the acculturation process (e.g., introduction of new religions) can affect the fishers’ worldview.

**Method:**

The selection of participants was done through intentional sampling. The content qualitative analysis was carried out to interpret the data from semi-structured interviews with 29 artisanal fishers.

**Results:**

The mythical representations that inhabit the aquatic environments in the surveyed area were as follows: *Mãe d’ água*, *Cabeça de cuia*, *Muleque d’água*, *Visage*, *Piratinga*, *Sucuiuiu*, and *Luz* e *Arco-íris*. These beings have distinctions regarding the form and attributions and can be associated with seasonality (temporal markers) and specific habitats (spatial markers). The respect and fear feeling of the mythical beings were striking among the fishers interviewed. For instance, we have record offering practices in order to obtain protection and success during the fisheries. These practices suggest that there may be local conservationist habits in fisheries management. However, the advancement of urbanization and the introduction of new religions that deny the existence of mythical entities are factors that can generate the acculturation process among the fishers.

**Conclusions:**

It is therefore necessary to carry out more studies in the surveyed area in order to evaluate the existence of possible patterns in the relationship between fisher and mythical beings. This information could confirm the role of mythical beings as environmental regulators. Consequently, it could be considered in the conservationist policies of fishery resources, reinforcing the importance of local knowledge and cultural factors for fishing management approaches.

## Background

The aquatic environments are sacred resources in all traditions throughout human history, relating directly to the people’s cosmology and culture [1]. Rivers, lagoons, and seas integrate the universal symbology of waters, which constitute various images and meanings, as well as manifested in sacred and mythological ceremonies or in the management of natural resources [2, 3]. These environments are linked to the worldview of many traditional societies, where there is an interconnection between the natural world, the supernatural, and the social organization [4].

Studies have identified an intrinsic relationship between traditional societies and spirits inhabiting aquatic environments around the world [5–8]. Such mythical beings are true guardians and owners of aquatic resources according to the worldview of these traditional people. Riverside dwellings respect and fear the waters and supernatural beings that they believe to live in these environments [9, 10]. This relationship implies a direct interaction of traditional people with exploited ecosystems and can contribute to the regulation of fishing activities, since a range of rules and taboos are incorporated into their beliefs and can contribute to the maintenance of these environments [8, 11].

Latin America high cultural and biological diversity plays an endless source of human-nature interaction studies [12–16]. In fact, Brazil has a growing number of researches on the wildlife exploitation dynamics by local populations, contributing for understanding these interactions and their implications for biodiversity conservation [17, 18]. Unsurprisingly, such researches’ approach is mostly utilitarian, which includes animal trade and use for food (especially bushmeat and fishing), zootherapeutic, magical/religious practices, and pet purposes [19–22].

On the other hand, Brazilian traditional societies’ wildlife management is linked to mythologies built over the centuries which constitutes their worldview [4, 23]. In Amazon, for example, animistic beliefs among indigenous people show that intentionality and reflexive consciousness are not exclusively human attributes, but also of other beings [24–27]. Similarly, this worldview has been observed among Amazon traditional non-indigenous people and in other regions of Brazil, such as Northeast fishing community [28–32].

Fishing communities has settled in main freshwater systems and alongside Brazilian coastal zone [33]. Artisanal fishing is an activity directly linked to the sociocultural issue and it is based on local knowledge in many places. This is particularly true in areas far from large urban and research centers [34]. In several aspects, local knowledge has contributed to the natural resources use and participatory management associated with the aquatic environments [35, 36]. However, this type of knowledge is threatened by the influence of new cultures to the detriment of pre-existing beliefs [8]. Consequently, local people are therefore at risk of losing some of their traditions [37], and studies are required to strengthening the collective memory of meaning for personal, spiritual, family, and welfare life [9].

The Brazilian northeast is particularly rich in coastal and continental aquatic ecosystems, highlighting the Parnaíba river basin. In fact, this basin harbors approximately 150 species of fishes [38], and it is important for housing an expressive contingent of fishers who live under strong interaction and dependence on fisheries resources [39]. These characteristics highlight the Parnaíba basin as a suitable *locus* for cosmological studies in traditional communities, for example, knowing how the supernatural beings that inhabit the freshwater systems inserted in fishing communities interact with fishers and fishes and how this worldview can imply the management of fisheries in the region.

The main goal of our research was to analyze the fishers’ worldview regarding the supernatural beings that inhabit the aquatic environments in the Amarante community, in Mid-North of Northeast Brazil. We analyzed how these mythical beings interact with fishers and fishing resources, as well as whether this interaction interferes with fishing behaviors in the region. This study also discusses fishers’ religiosity aspects and presence of community events involving the elements of their worldview.

## Methods

### Study site

**Fig. 1:**
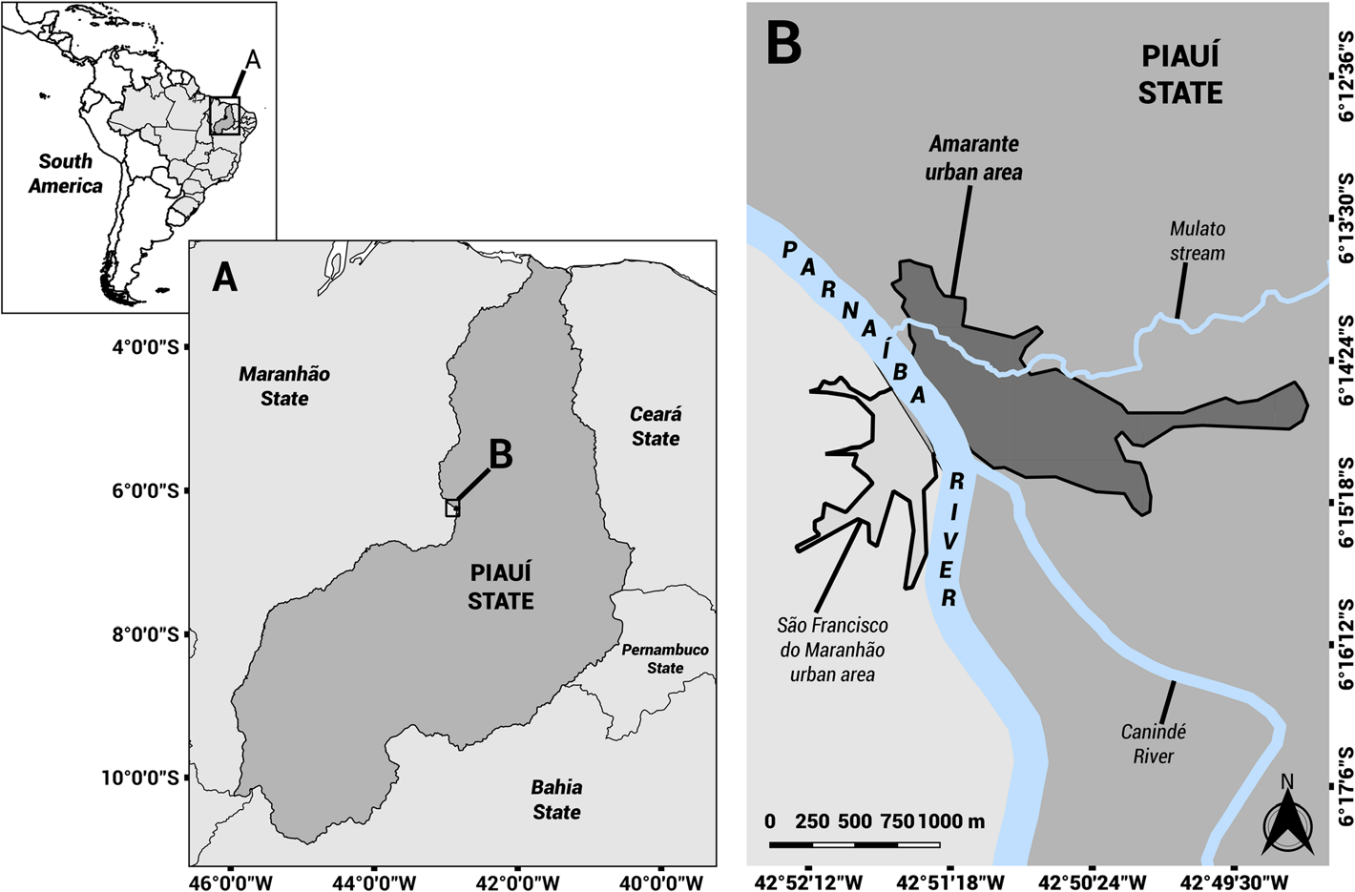
Location of the Amarante community, in mid-west region of Piauí, Northeast Brazil. Source: [39]

The Amarante community (6° 15′ 2″ S, 42° 51′ 12″ W) is located in Parnaíba River middle course, in the mid-west of Piauí State (Fig. 1). The Acoroá and Gueguê indigenous people populated this region by the end of the seventeenth century, when the European colonizers arrived and initiated successive territorial conflicts that led to the natives’ dispersion. The commercial flow formed on the Parnaíba banks led to the creation of São Gonçalo port in the first half of the nineteenth century. This locality became a regional headquarters in 1861 with the expansion of the river trade, and it was named as Porto de São Gonçalo de Amarante, which in the following decade rose to the category of Amarante town [40].

Currently, the population of Amarante is about 15 thousand inhabitants, with gross domestic product per capita of R $7198.74 in 2015 (US $1833.75), and has low Human Development Index (0.598) [41, 42]. These inhabitants depend on varied economic activities, linked to urban area (e.g., services provision) and countryside (e.g., crop cultivation, forestry, and livestock farming) [42].

The fishery also plays an important activity for the Amarante inhabitants. According to local fishing associations (Piauí State’s Fishers Federation Z-3 Colony, and the Union of Fishermen and Fisherwomen of Amarante and Francisco Ayres of Piauí and São Francisco do Maranhão – SindPesca), there are about 200 fishers living only in the urban area. The fishing activity is developed throughout the year, with restrictions from 15 November to 16 March, period in which a fisher can fish up to 5 kg of fish plus one specimen per day (see Brazil Environmental Ministry Normative Ruling No. 40-2005).

The main Amarante Rivers are the Parnaíba and Canindé. These rivers have a variety of habitats that are annually influenced by the seasonal water pulse of the region (alternating between rainy and dry periods), as well as the flow of the Boa Esperança Hydroelectric Power Plant. The climate is tropical with rainy monsoon (Am) in the Köppen classification, with average annual precipitation between 1300 and 1600 mm [43].

### Data collection and analysis

The methodological design of this study was based on the systemic approach [44]. This procedure consists of recording and analyzing the maximum perception of a phenomenon regarding the multiple dimensions, whether sociability, cultural, economic, political, or religious. We collected the data between February and March 2017. The selection of participants of this study was intentional, from the identification of key informants among the older fishers and subsequent application of the snowball technique [45].

Data were obtained through semi-structured interviews and the non-member participant observation technique [45, 46]. This observation consisted of accompanying and documenting textually or with photographs socio-cultural activities developed by fishers without the involvement of the interviewer. The questions addressed the following key points: cosmological (mythical beings that inhabit the rivers in the Amarante territory and whether they influence the use of fishing resources) and socioeconomic aspects (age, gender, schooling, marital status, family size, fishing experience, and living time in the surveyed area), as well as questions about religiosity and ceremonies linked to fisheries.

Interviews were conducted by a single researcher to reduce sampling errors, and a trust relationship (*rapport*) was also established to ensure greater data reliability [47]. We performed a qualitative content analysis in order to interpret the interview data [48]. This technique consisted of transcribing, organizing, and systematizing the collected data.

## Results

A total of 29 fishers participated in this research (23 men and six women) aged between 27 and 90 years (mean ± standard deviation: 52.83 ± 14.94). The fishing experience and dwelling time at surveyed area varied from 17 to 84 years (41.93 ± 14.64) and 15 to 90 years (45.17 ± 17.20), respectively. Table 1 summarizes the socioeconomic characteristics of the interviewees.

**Table 1:** Socioeconomic aspects of interviewed fishers from Amarante, mid-west region of Piauí, Northeast Brazil

		*n*
Age group	Less than 41 years old (years old)	06
	41–60 years old	17
	61 or older	06
Schooling	Very low (illiterate or semi-literate)	03
	Low (incomplete or complete elementary school “ensino fundamental”)	22
	Medium (incomplete or complete secondary school “ensino médio”)	04
Marital status	Single	04
	Married or stable union	22
	Divorced or separated	01
	Widower	02
Family size	Less than 4 people (p.)	15
	4–6 p.	12
	7 p. or more	02
Fishing experience	Less than 31 years (years)	08
	31–50 years	14
	51 years or more	07
Living time in the surveyed area	Less than 41 years (years)	13
	41–60 years	12
	61 years or more	04

### Fishers’ worldview: man/supernatural connection

The Amarante aquatic ecosystems are inhabited by supernatural beings according to the interviewed fishers’ worldview: “has more eyes in the water than hair on the earth. It has a lot of creatures in the water” (Int. 9).

#### *Mãe d’água*

The *Mãe d’água* was the mythical entity regarded as “the queen of the waters” and responsible for safeguarding the water sources, as well as the natural resources found in this environment, as the fishes and even the fishers: “(...) she assures the waters not to let the river die” (Int. 13); “(...) guardian of the waters; she lived in a place that never dried up” (Int. 3); “owner of the fishes, owner of the waters; releases the fishes for them to leave; If she does not release it does not catch any” (Int. 12); “(...) only catches the fish if she allows (...) protector of the fishes living in the water” (Int. 8). “She protects the fisher” (Int. 4).

The reports indicate that the *Mãe d’água* has part human part fish: “(...) like a woman; human on top and fish underneath” (Int. 5); “(...) more fish type” (Int. 2); “(...) transformed into a woman, part fish (low – flap, fish rudder)” (Int. 15). “(...) Woman, big hair, black (...)” (Int. 9); “[uses] black hair to the waist (...) long dress, pink with white flower and long sleeve” (Int. 11).

A fisher said one of the ways to get lucky in fishing is by acquiring the comb that the *Mãe d’água* possesses: “(...) says she has a comb and the person who catches is sacred/blessed and every time they arrive in the river there is a fish”. This fisher reported the story of another fisher who got the *Mãe d’água* comb: “once the *Mãe d’água* jumped into the water and left the comb. She asked for the comb and spoke [the fisher] to [her] first release the fish. Since then this fisher has been lucky in fishing” (Int. 9).

The *Mãe d’água* is respected and feared on the part of the riverine, being the fear related to her singing: “heard the song of her, which the fishers are enchanted and are attracted to the deep waters” (Int. 8); “(...) had a singing (...) say that the *Mãe d’água* took a girl” (Int. 16). One interviewee reported that he was once on the Parnaíba River on the late shift and in the rainy season in his canoe when he saw a woman who asked him: “do you know the queen of the waters?” Then, she jumped into the water and took the interviewee’s canoe.

One of the ways to please the *Mãe d’água* is by giving her offerings (e.g., flowers, alcoholic beverages, and light candle). People who do not fish in order to obtain advantages also do this practice: “(...) it gives people luck, to have things, to be happy, to the couple. In New Year’s Eve the people give offerings, light candle (...)” (Int. 10).

#### *Cabeça de cuia*

The *Cabeça de cuia* was described as a mythical entity of anthropomorphic traits: “big head, human body, small, black” (Int. 14); “big head, black, boy, small eye” (Int. 10); “black, red eyes, bad hair” (Int. 9). His origin is counted in different ways, but all the interviewees’ reports converge, basically, to the story of a son who was cursed by his own mother, as reported by a fisher:

There was an old woman who liked a son (...) he was very fond of fishing. The fish he liked the most to fish was the ‘piau-curimatá’ and the ‘piau-coco’ (...) every fish he fished (...) only he who would eat its head. One day he took some big ‘piaus’, [the mother] took three to prepare and wanted to eat the head of the fish. When the son came home, he went to eat and he saw that the fish had no head he said: “my mom, who eat the head of this ‘piau’? Have I not said that all the fish I catch, all of them who have to eat the head are not me?” She said: “my son I eat it, because I wanted very much”. He said a few things to her that replied: “I’m not going to eat the fish head anymore, but when you realize you will have become a beast in the river that eats only the fish head”. Then he took the ‘coité’, gave a moan, ran out towards the river and never came back (Int. 1).

Like the *Mãe d’água*, the *Cabeça de cuia* has an intrinsic relationship with the waters, being seen by the fishers more during the rainy season in which the water level of the rivers is high. Additionally, the reports point out that the *Cabeça de cuia* is related to specific habitats, such as areas of confluences between different water bodies, backwaters, and riverbeds: “it appeared in the first water, in the first rains, that the river filled” (Int. 4); “(...) at that time the river was full, he stood in the middle of the river” (Int. 7); “(...) when the river is too full he likes to appear, in the middle of the river” (Int. 9). This mythical entity also interacts with the fishes as a protector and controller of this aquatic resource: “he defends the fishes” (Int. 7); “only catches the fish if he allows it” (Int. 8).

The fishers also reported offering practices to the *Cabeça de cuia* to succeed in the fishery: “the people put cigars on the bow of the canoe, the cigar disappeared and the fishery worked; in the other day the ‘piratinga’ was on the hook” (Int. 7). On the other hand, this myth is also feared: “(...) an old man cut the hand of the *Cabeça de cuia* and shortly afterwards died” (Int. 7); “(...) scares the fishers. One caught in the middle of the canoe, wanting to sink; the fisher tried to hit with the paddle in his hand but did not hit him” (Int. 10); “the fisher knew he existed, he was afraid and he ran” (Int. 4).

#### *Muleque d’água*

The reports show a similar descriptive very close between the *Muleque d’água* and the *Cabeça de cuia*, both being considered the same myth for some of the interviewed fishers. The *Muleque d’água* is considered “the son of the waters” (Int. 2) and has been described as an anthropomorphic form of short stature and dark skin (“black creature”, Int. 9). Like the entities described above, this entity is related to the fishes: “I think there is something between the *Muleque d’água* and the fishes (...) he gives you lucky when you put cigars on the bow of a canoe” (Int. 9). According to reports, however, this entity also attacks the fishers: “If he [the fisher] moved [with this entity], he [the entity] would turn the fisher’s boat (...) it was to kill” (Int. 3); “says he catches people” (Int. 6).

#### *Visage*

*Visage* is “night thing” (Int. 8). It is usually attributed to a deceased fisher, perceived in situations in which the fisher hears noises such as a cast net, dive, or tree falling into the water: “the elders say they were fishers who died drowned” (Int. 15); “people who fished and died or drowned people” (Int. 14); “(...) heard the noise of a cast net but had nothing; perhaps the soul of an old fisher who died” (Int. 5); “it was a noise similar to a fisher’s cast net, but it was no one” (Int. 13); “(...) sound of falling tree, but saw no any wave” (Int. 14); “noise echoing in the hills” (Int. 17). In these situations, fishers withdraw from the site to fish in other areas for fear or respect.

The reports indicate that the *Visage* is also associated with the rainy season: “where there is large water, the *Visage* appears” (Int. 13). This mythical entity also is related to habitats such as backwaters and confluences. For example, a fisher reported that Remansão, Remanso da Arara, Pedrinha, Barra da Tapera, Purga, and Boa Vista are susceptible sites for to see *Visage*.

#### *Piratinga* and *Sucuiuiu*

The natural aquatic environments of Amarante are also inhabited by myths that possess zoomorphic traits. *Piratinga* is a myth attributed to big fish and is feared by fishers: “(...) very enormous that pulled the canoes” (Int. 15). These fishes were seen in stretches of the Parnaíba River, in the sites Purga and Morro da Arara, as well as in an underground cave under the Catholic Church in the Amarante urban area: “the house is in the river; when she was moaning, she rocked the church, sometimes cracked the walls” (Int. 11).

Another myth that possesses animal form is the *Sucuiuiu*, a snake very feared by fishers able to influence in the decision making of the fisher during the fisheries, such as how to change the fishing place: “a creature that has seen and who is afraid” (Int. 4); “false beast, if he appeared we had to seek another place to fish” (Int. 11).

#### *Luz* and *Arco-íris*

Physical phenomena of nature are also part of the cosmological imagery of the Amarante’s fishers. The *Luz* was described as similar to fire, of blue or red color, and observed by fishers during generally night fisheries: “(...) kind of fire that was clearing everything that appeared; flew, vanished; when there was winter, at the foot of the mountain; (...) was not fixed; ran, went out and left suddenly” (Int. 2); “it appears during the night; light fire; (...) it appears to lone fishers; the oldest in respect was silent” (Int. 8). An interviewee said that the *Luz* is “like a flying saucer” (Int. 10), which saw it landing in the Serra de Santa Cruz, on the left bank of the Parnaíba River, in São Francisco do Maranhão town.

The *Arco-íris* is another phenomenon of nature that, according to reports, has the ability to transport fish, taking them from one place and placing them in another: “take the fish from one place and take it to another” (Int. 8). This ability to appear and disappear mysteriously makes fish beings enchanted in the fishers’ worldview: “the report of the oldest says that the fish is enchanted. It appears a time and disappears; the Canindé River is dry, if it fills today, the fish appears; so he has it as enchanted” (Int. 8); “the fish is enchanted, he flees from the sight of us; There are people with bad eyes, the fish does not let everyone fish it” (Int. 11).

The fisher tends to avoid feelings of ambition or greed over the fishes to succeed in the fishery: “(...) is enchanted because of the great eye; if you have greed, the big eye does not catch fish. Before fishing, the person cannot ask the fish for the fisher” (Int. 14); “(...) if anyone asks before, if the fishers receive money in advance, they have no luck in the fishery; go out in silence (...). If you start admiring, the fish disappears” (Int. 15).

However, fisher’s worldview in which mythical entities inhabit the aquatic environments of Amarante and that part of these are protectors of water and fish was not observed among all interviewees. For example, a fisherwomen considered such mythical beings as “trancoso” history, understanding them as something that “does not exist or existed and no longer exist” (Int. 4).

### Religiosity and festivities related to fisheries

Catholicism was the most representative religion among respondents, including from devotees of saints (e.g., Saint Lucia) to those who do not participate in religious activities. On the other hand, the Protestants accounted the minority along with the fishers who admitted having the habit of going (or have already been) in cultural centers of veneration of saints and deities from Afro-Brazilian beliefs.

Fishers consider nature as something fundamental to be experienced in everyday life, which is why they say they love and feel happiness in exercising artisanal fishing. This activity is considered therapeutic, providing distraction and calming in face of the problems faced in day-to-day life. For example, there were reports of fishers who forget about family problems when they are fishing in the river. In addition, fishers feel proud of their profession, especially after the creation of local fishing associations.

The fishers reported that they were discriminated by Amarante’s elite before the local fishing associations existed. For example, a fisher reported that it was humiliating to make a bank loan, because they had to search for third parties (e.g., property owners) to represent them as a guarantor, and that in the present day, it is possible to make direct loans by the fishing association. Furthermore, a fisherwoman said that she was formerly “criticized and mocked” by people when she was “smelling like fish”.

Local fishing associations are important for the promotion of festivities among fishers. The largest traditional festival of the Amarante’s fishers is the Canoe Rowing Race. This event occurs annually and consists in a march from SindPesca’s headquarters to the pier on the banks of the Parnaíba River called Cai N’água. Then, there are competitions of various modalities among fishers (e.g., canoe regatta, scuba diving, beach running, fishing nets), with awards. The party extends until the night shift with performances by local and regional music groups. In addition to this event, the fishers also celebrate commemorative dates, such as the anniversary of the associations, and their members, as well as mother and father days.

## Discussion

### Water spirits: forms and attributes according to fishers’ worldview

The waters’ symbolism is made up of myths whose anthropomorphic, zoomorphic, or both date back thousands of years, as observed in the Greek, Chinese, and Austro-Asian aquatic mythologies [49]. In Africa, for example, Congo Basin traditional population attribute anthropomorphic or zoomorphic traits to water spirits [10]. On the other hand, these beings can be represented by large snakes or plants, as observed between indigenous and non-indigenous people in the Brazilian Amazon [26, 50]. Among the Mapuche indigenous people, in Patagonia, the myth *Ngen*, for example, can take on different forms, including a woman with a fish body [7].

There are also reports of mythical beings who have similar forms to “fireballs.” In northeastern Brazil, these phenomena are known in fishing communities of Alagoas, called the *Fogo Corredor* [28], and Bahia, with the name *Biatatá* or *Bate Facho* [51]. Similarly, this type of myth also was observed among the Amarante fishers, who reported about the existence of light in the rivers.

In general, the Brazilian myths and the diversity of cultural representations come from the convergence of different cultures. For instance, the *Mãe d’água* results from the aggregation of the indigenous (*Iara*), African (*Iemanjá*), and European (*Sereia*) mythological universe [52, 53]. This mythical entity is widely known among fishers of Amarante, as well as other regions of Northeast Brazil [28, 29, 54, 55], where they are seen as a protector of nature. Similarly, the Kakuna indigenous people, in the Peruvian Amazon, consider many aquatic beings as “water mothers,” who are responsible for the existence of water, which is why the disappearance of these myths is a concern for these people [56].

The myths are feared by many fishers, although they are responsible for the protection and supply of fish, and their stories about causing harm to people, and even killing them, date back to antiquity [49]. In the present day, reports similar to this were observed among the Amarante fishers, where they are attributed to the myths *Mãe d’água*, *Cabeça de cuia*, and *Muleque d’água*, as well as in various fishing communities around the world [6, 7, 56]. In the Brazilian northeast, *Nego d’água* and *Caboclo d’água* derive from the same myth (*Compadre d’água*), which is considered a bad element and the maximum authority of the São Francisco River basin [54]. In this basin, there is a record that the *Nego d’água* steals fish from the fishers’ canoe [28], similarly to what the Amarante fishers mentioned about the *Muleque d’água* in the rivers of this region. The *Caboclo d’água*, which also has a record among fishers in the Brazilian Amazon, is considered for these fishers as dangerous and, therefore, is feared [30].

### Fishers’ worldview: implications for fisheries management

The ambivalent feeling of fear and fascination towards the waters, as well as the practices of cultivating aquatic deities, date from antiquity [49]. Nowadays, these mythical representations are observed in the traditional societies’ worldview around the world, especially among people whose belief is based on the animistic perspective [8, 24, 26]. This perspective consider that there is no separation between the physical and spiritual worlds, where other beings besides humans, such as animals and plants, also possess souls, forming a hierarchy in which man is not at the top of this system [25].

This is particularly true in case of genius loci (spirits of place) regarding the human–nature interactions [57]. Such connectivity has contributed to conservationist practices based on local traditional knowledge [58]. In Africa, for example, the Lake Nokoué people’s belief in Voodoo spirits contributed to the formation of a set of rules and taboos among the artisanal fisheries, including the establishment of sacred areas where fishing is prohibited, protecting fish spawn sites, and limitation of fishery production [8, 59].

Local practices among traditional people also include launching herbs, coffee beans, cassava-based alcoholic beverages, among other offerings, in rivers [10, 49]. The Mapuche Indians, in South America, also follow a cultural protocol of entering the waters or managing their resources, leaving payment in coin or something vegetable as offerings to the water’s spirits [9]. Similarly, the riverine use cigars or “beiju” (cassava-based food) in the river in order to conquer the *Nego d’água*, in the Brazilian Northeast [53]. These practices were also observed among the Amarante fishers, suggesting that they can make decisions based on respect or fear of the waters’ mythical beings during the fisheries, as in the cases where the fishers stopped fishing in places where they had direct experiences with supernatural beings.

However, the socio-economic modernization and the introduction of new institutions and religions are replacing and damaging the traditional management of resources [8]. Consequently, this interferes negatively in the pre-existing conservationist principles in the local belief [60]. In this sense, it is possible that an acculturation process among the Amarante fishers is underway in this region, especially among recent generations, considering the advancement of the urbanization and the introduction of new religions that deny the existence of mythical entities. This conjecture is aggravated by the advancement of strategies and fishing technologies that do not consider the ancestors’ culture, although attenuated by typical cultural manifestations of the region. This could imply a change or disappearance of mythological thought originally established in the Amarante community and, consequently, in the conservation of fisheries resources in this region.

## Conclusions

The waters are intimately linked to the Amarante fishers’ worldview, especially among the most experienced, who recognize the existence of mythical beings who live in association with rivers and their natural resources, such as fish. These beings have distinctions both in relation to the form and in assignments and may be associated with distinct seasonal periods (temporal markers) or specific habitats (spatial markers).

The feeling of respect and fear regarding to mythical beings suggest that there may be local conservationist habits in fisheries management based on the worldview in which mythical beings act as regulators of this activity. However, the advancement of urbanization and the introduction of new religions that deny the existence of mythical entities are factors that can generate the acculturation process among the Amarante fishers, especially among recent generations.

It is therefore necessary to carry out more studies in the surveyed area in order to evaluate the existence of possible patterns in the relationship between fisher and mythical beings. This information could confirm the role of mythical beings as environmental regulators. Consequently, it could be considered in the fishery resources conservationist policies, reinforcing the importance of local knowledge and cultural factors for fishing management approaches.

## Data Availability

The datasets used and/or analyzed during the current study are available from the corresponding author on reasonable request.
